# COVID-19: asymptomatic carrier transmission is an underestimated problem

**DOI:** 10.1017/S0950268820001235

**Published:** 2020-06-11

**Authors:** Hongjun Zhao, Xiaoxiao Lu, Yibin Deng, Yujin Tang, Jiachun Lu

**Affiliations:** 1 State Key Lab of Respiratory Disease, Institute for Public Health, School of Public Health, The First Affiliated Hospital, Guangzhou Medical University, Guangzhou, China; 2 Department of English and American Studies, Faculty of Languages and Literatures, Ludwig Maximilian University, Munich, Germany; 3 Department of Infectious Disease, The Affiliated Hospital of Youjiang Medical University for Nationalities, Baise, China

**Keywords:** Asymptomatic carrier, COVID-19, infectivity of asymptomatic, proportion of asymptomatic, SARS-CoV-2

## Abstract

At the present time, COVID-19 is spreading rapidly [1]. The global prevention and control of COVID-19 is focused on the estimation of the relevant incubation period, basic reproduction number (*R*_0_), effective reproduction number (*R*_t_) and death risk. Although the prevention and control of COVID-19 requires a reliable estimation of the relevant incubation period, *R*_0_, *R*_t_ and death risk. Another key epidemiological parameter-asymptomatic ratio that provides strength and range for social alienation strategies of COVID-19, which is widely defined as the proportion of asymptomatic infections among all disease infections. In fact, the ratio of asymptomatic infection is a useful indicator of the burden of disease and a better measurement of the transmissibility of the virus. So far, people have not paid enough attention to asymptomatic carriers. The asymptomatic carriers discussed in this study are recessive infections, that is, those who have never shown symptoms after onset of infection. We will discuss three aspects: detection, infectivity and proportion of healthy carriers.

## Asymptomatic carriers were reported

**Fig. 1.**
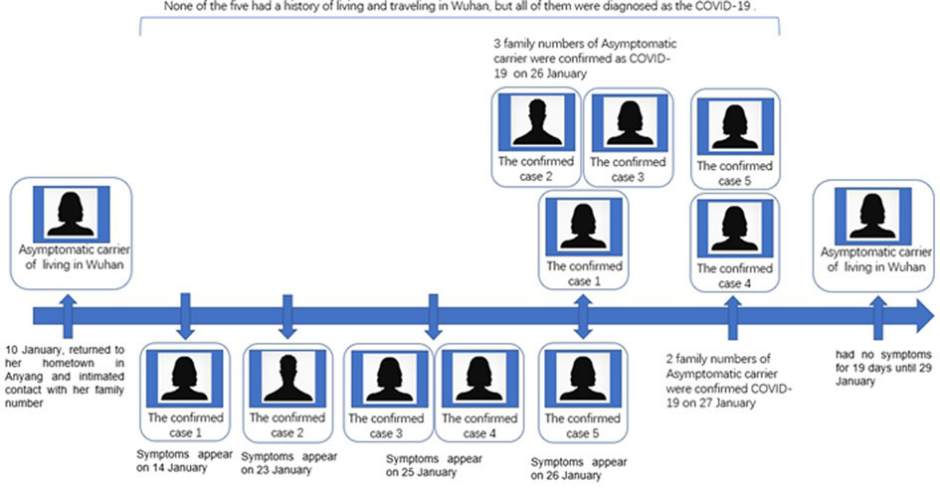
Timeline of the onset of symptoms (fever, pharyngalgia, chest tightness and so on) and asymptomatic carrier of SARS-CoV-2 that causes COVID-19 in a familial cluster.

On 27 and 28 January 2020, the Anyang Centre for Disease Control and Prevention reported a familial cluster of cases with COVID-19, which may be caused by an asymptomatic carrier in Anyang, Henan province, China [2–4]. The timeline of the onset of symptoms and asymptomatic carrier of SARS-CoV-2 that causes COVID-19 patients in a familial cluster is shown in Figure 1. On 10 January 2020, the index case, who lived in Wuhan, returned to her hometown in Anyang city to celebrate the Spring Festival with her family. This familial cluster of six cases (index case, and cases 1−5) was infected with COVID-19, and only the index case had been to Wuhan, and then her family members started to get sick one after another. However, the index case showed positive RT-PCR test results but no symptoms and normal chest computed tomography (CT) for 19 days before January 29, which is comparatively long but still within the reported maximum incubation period of 24 days [5]. Case 1(index case's aunt) is the earliest confirmed case in Anyang city and the only case in district A of the city. Case 1 has neither links to Wuhan nor having any history of contact with confirmed cases in the two weeks before onset, only a history of close contact with index case, who has become the only evident source of infection. Similarly, the other four cases (cases 2−5) had no epidemiological exposure in the 14-day period prior to onset, except the contact with index case, and case 2 (index case's father) and case 5 (index case's mother) are the earliest two cases in district B of the city. Therefore, the asymptomatic carrier had a relevant epidemiological history and it may be concluded that there is an asymptomatic spreader of COVID-19.

Bai *et al*. reported this familial cluster of cases and presumed there is a transmission of SARS-CoV-2 by an asymptomatic carrier in Anyang on 21 February 2020 [6]. Zhang *et al.* reported a familial cluster of five cases that was infected with COVID-19 in Beijing on 27 March 2020. Zhang *et al.* showed that the index patient remained asymptomatic for 42 days, which was much longer than the reported maximum incubation period (24 days) [7]. These two family clusters of COVID-19 cases raised concerns about asymptomatic carrier transmission.

## Infectivity of asymptomatic carriers

Chen Yi et al. compared the infectivity between asymptomatic carriers and symptomatic cases [8]. The study followed up on the close contacts of 59 imported infectious patients (51 symptomatic cases and eight asymptomatic carriers). A total of 2147 close contacts of these individuals were investigated, and among them of 132 local residents were infected (110 symptomatic cases and 22 asymptomatic cases). It was found that the total infection rate was 6.15% (132/2147), and the infection rates of close contacts of symptomatic and asymptomatic infections were 6.30% (126/2001) and 4.11% (6/146), respectively, with no statistically significant difference (*P* > 0.05). This was the first published quantitative study on the infectiousness of the asymptomatic transmission. Wu *et al.* [9] inferred that 126 cases (51−126) were transmitted from the symptomatic cases, while six cases (8−6) were transmitted from the asymptomatic cases. On average, each symptomatic patient could transfer the virus onto nearly three other individuals, while each asymptomatic carrier infected less than one other individual [9]. In other words, the transmission efficiency of the asymptomatic carrier was about 1/3 of that of the symptomatic case. However, it showed that asymptomatic infected cases with SARS-CoV-2 is highly contagious and transmitted through close contact. It is worth noting in the study that the estimation of asymptomatic transmission, according to Chen Yi *et al.* [8] and others, was based on information of close contacts who were tracked, which effectively blocked the spread of the disease through medical isolation and cutting off the transmission. Therefore, the results may be inconclusive and does not reflect all characteristics of asymptomatic spread in the real world, and the infectivity of this mode of transmission may be underestimated. The report by Chen Yi et al. had some limitations. Firstly, the infectivity was not quantified and needed more research to verify, and it was a single study with the absence of interval estimation. Secondly, it had insufficient estimates of the infectiousness of asymptomatic cases, secondary attack rate can more accurately reflect the infectiousness.

## Proportion of asymptomatic carriers

Chen Yi *et al.* reported that asymptomatic infections accounted for 15.7% (30/191) of the total number of infections [8]. Japanese researchers investigated the cases on the Princess cruise ship, applied the statistical model with time-series dataset, and estimated the proportion of asymptomatic carriers in all infected cases to be 17.9% (95% CI 15.5–20.2%) [10]. The researchers believed that their analysis underestimated the proportion of asymptomatic cases, the reasons are as follows. To begin with, most of the passengers were over 60 years old, the nature of the age distribution may lead to underestimation. Next, tests by RT-PCR were conducted with only symptomatic patients in focus at the early phase of the quarantine, and asymptomatic carriers were left out as a result of this. In addition, Japanese scholars estimated that the proportion of asymptomatic patients among infected individuals who were Japanese citizens and were evacuated out of Wuhan was 30.8% (95% CI 7.7–53.8%) [11]. There are currently three estimations of the proportion of asymptomatic cases in the whole infected population. The proportion might be much higher than the proportion of asymptomatic patients (1.0–1.2%) estimated by the majority of researchers estimated through clinical studies and other surveys conducted in the early stages of the epidemic [12, 13]. It should be noted that the proportion of the asymptomatic reported in most early studies comes from the proportion of existing test results, not from the proportion of the asymptomatic, so it is impossible to accurately estimate the asymptomatic infection rate. If the estimation of Japanese scholars are in line with reality, then our current proportion of asymptomatic cases is seriously underestimated. Similar to norovirus, published studies have reported that up to 30.0% of norovirus infection remains asymptomatic [14, 15], while such asymptomatically infected individuals are known to excrete substantial volume of viruses too [16].

In summary, there is evidence that COVID-19 asymptomatic carrier can transmit SARS-CoV-2, and its infectivity is similar to that of symptomatic patients. So, considering the similarity of reported viral load between the asymptomatic and the symptomatic patients [17] and a relatively high proportion of the asymptomatic cases, the asymptomatic carriers may jeopardise efforts to contain COVID-19 transmission in public health. Because the proportion of the asymptomatic cases is underestimated and the infectiousness and prevention measures of the asymptomatic have not attracted enough attention, there will be a critical flaw in prevention and control of COVID-19. Thus, the danger of asymptomatic spread should arouse public awareness and more scientific attention into researching asymptomatic transmission in order to contribute to developing more scientific prevention and control strategy and overcoming the epidemic as soon as possible.

## References

[1] World Health Organization. Novel Coronavirus (2019-nCoV) Situation Report-89. Available at https://www.who.int/docs/default-source/coronaviruse/situation-reports/20200418-sitrep-89-covid-19.pdf?sfvrsn=3643dd38_2.

[2] Anyang Center for Disease Control and Prevention (2020) Epidemic situation report. Anyang daily, 27th Jan 2020.Available at https://weibo.com/ayrb?is_hot=1#_loginLayer_1580375657980.

[3] Anyang Center for Disease Control and Prevention (2020) Epidemic situation report. Anyang daily, 28th Jan 2020. Available at https://weibo.com/ayrb?is_hot=1#_loginLayer_1580375657980.

[4] Henan Municipal Health Commission (2020) Report of novel coronavirus infected pneumonia in Henan Province. Henan Municipal Health Commission.

[5] Guan WJ (2020) Clinical characteristics of coronavirus disease 2019 in China. The New England Journal of Medicine 382, 1708–1720. 32109013 10.1056/NEJMoa2002032 PMC7092819

[6] Bai Y (2020) Presumed asymptomatic carrier transmission of COVID-19. Journal of the American Medical Association 323, 1406–1407. 10.1001/jama.2020.2565 PMC7042844 32083643

[7] Zhang J (2020) Familial cluster of COVID-19 infection from an asymptomatic. Critical Care 24, 119. 32220236 10.1186/s13054-020-2817-7 PMC7100442

[8] Chen Y (2020) The epidemiological characteristics of infection in close contacts of COVID-19 in Ningbo city. Chinese Journal of Epidemiology 41, 668–672. 10.3760/cma.j.cn112338-20200304-00251 32447904

[9] Wu Z (2020) Asymptomatic and pre-symptomatic cases of COVID-19 contribution to spreading the epidemic and need for targeted control strategies. Chinese Journal of Epidemiology 41, 0–0. 10.3760/cma.j.cn112338-20200406-00517 32274917

[10] Mizumoto K (2020) Estimating the asymptomatic proportion of coronavirus disease 2019 (COVID-19) cases on board the Diamond Princess cruise ship, Yokohama, Japan, 2020. Eurosurveillance 25, 2000180. 10.2807/1560-7917.ES.2020.25.10.2000180 PMC7078829 32183930

[11] Nishiura H (2020) Estimation of the asymptomatic ratio of novel coronavirus infections (COVID-19). International Journal of Infectious Diseases 94, 154–155. 32179137 10.1016/j.ijid.2020.03.020 PMC7270890

[12] Wu Z (2020) Characteristics of and important lessons from the coronavirus disease 2019 (COVID-19) outbreak in China: summary of a report of 72314 cases from the Chinese Center for Disease Control and Prevention. Journal of the American Medical Association 323, 1239–1242. doi: 10.1001/jama.2020.2648. 32091533

[13] Novel Coronavirus Pneumonia Emergency Response Epidemiology Team (2020) The epidemiological characteristics of an outbreak of 2019 novel coronavirus diseases (COVID-19) in China. Chinese Journal of Epidemiology 41, 145–151.32064853

[14] Phillips G (2011) Risk factors for symptomatic and asymptomatic norovirus infection in the community. Epidemiology & Infection 139, 1676–1686. 21205382 10.1017/S0950268810002839

[15] Miura F (2018) Estimating the asymptomatic ratio of norovirus infection during foodborne outbreaks with laboratory testing in Japan. Journal of Epidemiology 28, 382–387. 29607886 10.2188/jea.JE20170040 PMC6111106

[16] Atmar RL (2008) Norwalk virus shedding after experimental human infection. Emerging Infectious Diseases 14, 1553–1557. 18826818 10.3201/eid1410.080117 PMC2609865

[17] Zou L, (2020) SARS-CoV-2 viral load in upper respiratory specimens of infected patients. The New England Journal of Medicine 41, NEJMc2001737. 10.1056/NEJMc2001737 PMC7121626 32074444

